# Private patient perceptions about a public programme; what do private Indian tuberculosis patients really feel about directly observed treatment?

**DOI:** 10.1186/1471-2458-10-357

**Published:** 2010-06-22

**Authors:** Lancelot M Pinto, Zarir F Udwadia

**Affiliations:** 1 Department of Pulmonary Medicine, P.D. Hinduja National Hospital and Medical Research Centre, Mahim, Mumbai

## Abstract

**Background:**

India accounts for one-fifth of the global incident cases of tuberculosis(TB). The country presently has the world's largest directly observed treatment, short course (DOTS) programme, that has shown impressive results and covers almost 100% of the billion-plus Indian population. Despite such a successful programme, the majority of Indian patients with tuberculosis prefer private healthcare, although repeated audits of this sector have shown the quality to be poor.

We aimed to ascertain the level of awareness and knowledge of private patients with tuberculosis attending our clinic at a tertiary private healthcare institute with regards to the DOTS programme, understanding the reasons behind their preference for private healthcare, and evaluating their perceptions and reasons for accepting or failing to accept directly observed therapy as a treatment option.

**Methods:**

A structured interview schedule was administered to private patients with tuberculosis at the P.D. Hinduja Hospital and Medical Research Centre, Mumbai, India between January 2006 to November 2007.

**Results:**

Only 30 of 200 patients (15%) were aware of the DOTS programme. After being explained what directly observed therapy was, 136 patients (68%) found this form of treatment unacceptable.183 patients (91.5%) preferred buying the drugs themselves to visiting a DOTS centre. 90 patients (45%) were not prepared to be observed while swallowing their TB drugs, finding it an intrusion of privacy.

**Conclusions:**

Our study reveals a poor knowledge and awareness of the DOTS programme among the cohort of TB patients that we interviewed. The control of TB in India will undoubtedly benefit from more patients being attracted to and treated by the existing DOTS programmes. However, directly observed treatment, in its present form, is considered too rigid and intrusive and is unlikely to be accepted by a majority of patients seeking private healthcare. Novel strategies and more flexible options will have to be devised to ensure higher cure rates without compromising patient choice.

## Background

Tuberculosis (TB) is the seventh most common cause of mortality worldwide [1], and Indian patients account for one-fifth of the global incident cases of TB; 1.8 million new cases occur every year [2] It is one of the commonest infectious diseases in India and has caused significant morbidity, mortality and untold suffering in this country over the decades. The Annual Risk of Tuberculosis Infection (ARI) has been estimated to be about 1.5-1.7%, the risk being significantly higher in urban areas [3]. The Revised National Tuberculosis Control Programme (RNTCP) was formally launched in 1998, directly observed treatment, short-course (DOTS) being the cornerstone of the programme. The programme is presently the world's largest DOTS programme, covering almost 100% of the billion-plus Indian population [4]. In the cohort of patients treated with DOTS, akin to the experience of other countries around the world, the programme has shown impressive results in various criteria: case detection, treatment outcomes, and lower rates of failure, default and death.

Despite critiques finding the treatment offered in the private health sector to be unsound, sub-standard and inconsistent, 50-70% of tuberculosis patients in India continue to prefer private healthcare [5-7]. These patients are not monitored by the RNTCP and therefore, do not benefit from the potential success rates offered by DOTS. The further success of TB control in India will significantly depend on attracting these patients, and comprehending the reasons for the inability of a successful programme to be considered as the health provider of choice by the masses.

Our study aims at analyzing the awareness and knowledge of private patients with tuberculosis attending our clinic at a tertiary private healthcare institute with regards to the DOTS programme, understanding the reasons behind their preference for private healthcare, and evaluating their perceptions and reasons for accepting or failing to accept directly observed treatment (DOT) as a treatment option.

## Methods

The study was conducted at a large private hospital in Mumbai, India where all attending patients pay out-of-pocket for consultations. The hospital caters to a large suburban population of Mumbai city, and has a busy chest department, that serves approximately 60 patients a day. The treatment of tuberculosis at the hospital involves consulting the treating physician on a flexible basis for monitoring and fresh prescriptions. Although private-public mixes (PPMs) have been successful in various studies across the country, the hospital is not a part of any public-private mix (PPM) project; hence patients are not offered DOT as a treatment modality.

200 consecutive adult patients with a diagnosis of tuberculosis who opted to visit one of the authors, a private chest physician, in the outpatients department of the hospital between January 2006 to November 2007 were enrolled in the study at the time of diagnosis. The study was approved by the "P.D. Hinduja national hospital and medical research centre institutional review board", which is the ethics committee of the hospital. A written informed consent was taken from the patients at the time of enrollment. There were no incentives offered for the enrollment, and patients could choose to be anonymous. All of the 200 patients who enrolled consented to being part of the study and were then recruited.

At their second follow-up visit, a structured interview schedule was used to administer a four-part questionnaire covering their demographic features, followed by an assessment of their knowledge, attitude and practice regarding the choices in the treatment of tuberculosis (Annex 1, for the detailed questionnaire, see Annex 3). The questionnaire was administered by a physician or medical personnel trained to understand the intricacies of the DOTS programme; patients were at liberty to ask questions on aspects that needed further elaboration. In the event that a patient was unaware of DOTS, the interviewer explained the tenets of the programme and then evaluated the patient's attitudes and acceptability. The explanation offered included a detailed elaboration of the five key elements of DOTS. On an average, each interview lasted 30 minutes.

## Results

The patient sample included in our study comprised predominantly middle-aged, economically productive individuals. Over 90 percent of interviewed patients had at least a secondary school education, which corresponds to a minimum of seven years of formal schooling. The median family income was significantly above the national average. The demographic characteristics of the patients are summarized in Table 1. The overall responses to the interview are summarized in Table 2.

When asked whether they were aware of a new strategy to fight TB called DOTS, only 30 patients (15%) replied in the affirmative. Only 1 of the 200 patients (0.5%) could expand the DOTS abbreviation accurately, while 5 patients (2.5%) were only vaguely aware of what DOTS entailed.

After being explained what DOT entailed, each patient was asked if and why he/she found this form of treatment acceptable. The majority of private patients (136 out of 200, 68%) found this form of treatment unacceptable; the commonest reason cited (80/200, 40%) was that three visits to a clinic per week were cumbersome, time-consuming and inconvenient. The other reasons cited were the perceived poor quality of treatment offered by services that involved the government (26/200, 13%) and the lack of privacy (6/200, 3%). When asked why they preferred a private TB specialist to a government TB clinic, 136 patients (68%) had reservations about the quality of healthcare offered in government hospitals, with a general lack of trust in government services, lack of attention offered to patients, long waits, poor hygiene and suspect quality of drugs being cited as reasons. 50 patients (25%) chose private care based on word-of-mouth experiences, past experiences or a referral from their family physician. Only 64 patients (32%) found DOT acceptable, the commonest reasons for perceiving it as a sound treatment option being ensured compliance (30/200 respondents, 15%), that it was offered free (13/200 respondents, 6.5%) and guaranteed a complete supply of drugs (6/200 respondents, 3%).

When asked whether they themselves would be prepared to visit a TB clinic as frequently as thrice a week to receive drugs, only 51 patients (25.5%) answered in the affirmative. However, when specifically asked their choice, 183 patients (91.5%) stated that they would still prefer buying the drugs themselves once a month. 90 patients (45%) were not prepared to have a doctor or nurse actually observe them swallow each dose of their TB drugs, claiming this amounted to an intrusion of their privacy. The commonest reason given for not wanting to be supervised was that they were responsible individuals and knew the importance of taking their drugs as advised (36/200 respondents, 18%). A few responses that, while standing out in terms of clarity, reflected the overall sentiment have been summarized in Annex 2.

## Discussion

The DOTS programme in India is a highly successful one, covering India's billion-plus population [4]. Despite the success of the programme, 60-88 percent of Indian patients with tuberculosis choose to bypass the public health system completely and consult a private medical practitioner for treatment [8]. Drug marketing surveys indicate that 94 million dollars worth of antituberculous drugs were purchased in India in the year 2006; approximately 75 per cent of these drugs were procured from the private sector [9]. These figures are not limited to the urban patients; a recent survey in the slums of Bangalore suggested that up to 72% of patients with pulmonary tuberculosis first approached a private healthcare facility for treatment [10]. India has a huge, unwieldy, and poorly regulated private medical sector with an estimated 10 million registered doctors, with a ratio of 1 doctor per 1000 population, far in excess of the WHO guideline of 1 in 3000. Around 50% of these are qualified and registered non-allopathic doctors practicing alternative systems of medicine such as homeopathy, ayurveda, and unani [11]. The TB prescribing practice of these doctors has been found to be erratic and the majority of prescriptions do not comply with standard guidelines [7]. India has the second largest population of multi-drug resistant tuberculosis (MDR-TB) patients in the world [12]. A recent study in Mumbai found MDR-TB prevalence rates to be 24% among newly diagnosed, previously untreated patients and rates of 41% in first-line drug failures[13]. Approximately 8.4 million dollars worth of second line antituberculous drugs were purchased in the year 2006; almost all of it by the private sector[9]. A study conducted by us found that among 106 practicing general practitioners at a large slum in Mumbai who participated in the questionnaire-based study, only 6 physicians could write a correct prescription for a newly diagnosed patient of tuberculosis [14]. There is little doubt that the poor prescribing practices of physicians in the private sector fuel the MDR-TB epidemic as well.

Knowledge and awareness of DOTS and its success would be the first step in dispelling a patient's stereotypic views of the public healthcare system. Our study highlights lacunae in public awareness of DOTS. Even amongst our cohort of economically productive, educated Indians, only one patient (0.5%) could expand the DOTS abbreviation, and 5 patients (2.5%) were vaguely aware of what DOTS entailed. Only 30 of 200 private TB patients (15%) were even aware of the government using a strategy called DOTS in the fight to control TB in India. Similar results were obtained at a pilot study of an information, education and communication (IEC) campaign in Delhi, India, that found the knowledge of the people concerning tuberculosis and DOTS to be grossly lacking. While the level of awareness and a change in help-seeking behavior did change after the pilot IEC campaign, the change was primarily among the literate and those from a higher economic stratum [15]. The demographic profile of our patients comprised predominantly literate, middle-income individuals. Even in such a cohort, in a metropolitan city like Mumbai with almost universal access to television, the knowledge regarding DOTS was dismal, highlighting the government's failure to increase public awareness through information campaigns. It is essential to involve and sensitize the private healthcare in India in the fight against TB. The DOTS strategy and implementation of new strategies are essential in fighting the disease. Several existing PPMs have performed well [16,17], and these augur well for further success of such programmes, contributing significantly to the control of TB in India, if instituted in a systematic and planned manner.

An overwhelming 91.5% of the patients interviewed preferred purchasing the drugs themselves instead of enrolling in the DOTS programme. Traveling to a treatment center thrice a week in the midst of busy schedules in a traffic congested city like Mumbai seemed impractical to the majority of patients. These reasons have been identified in other studies as impediments to patient acceptability [18,19]. Moreover, the risks of transmission of TB in crowded waiting rooms at DOTS centres and the apprehensions of the same have to be acknowledged and should encourage the development of innovative strategies such as family-member observed DOTS, which is an inexpensive strategy that has been shown to be successful [20]. Most of the patients in our study, being literate and knowledgeable about the grave nature of their disease, considered themselves responsible enough to not need supervision while consuming medications, and therefore, did not find the prospect of consuming medications under supervision acceptable. Till the time that patients perceive being supervised as equated to being considered irresponsible, this is unlikely to change.

"Can I afford free treatment" was the provocative question asked by Lonroth et al. in a study analyzing the preference for private healthcare among patients with tuberculosis in Ho Chi Minh City, Vietnam [21]. The patients in our study seem to reflect a similar sentiment, with perceptions of the public healthcare system being predominantly negative, and indicative of a lack of trust in the care offered; the risk of being treated inappropriately being perceived as too high to offset the lure of free treatment. Such perceptions warrant serious introspection on identifying shortcomings and analyzing ways of improving the brand equity of services that involve the public sector.

A social assessment study submitted to the Central TB Division, Ministry of Health and Family welfare, Government of India, found that even among poor, marginalized groups consisting tribals, industrial workers, slum dwellers, and migrant populations, Government health services were sought as a last resort, often when financial resources had been exhausted by treatment by private practitioners, or the expensive laboratory tests ordered by the private practitioners. The reasons cited for preferring private care were proximity (and therefore, costs saved on transportation), flexibility and better attention given [22]. A thorough Pubmed search, coupled with a search of Indian medical journals failed to come up with a similar study offering an in-depth analysis of the knowledge and attitudes among urban TB patients affording of private health care toward healthcare offered through the public sector via the DOTS programme. Our study aims to fill this void and offer a perspective on why so many patients prefer to go private.

A shortcoming of our study is that the demographic profile of the patients included is not representative of all tuberculosis patients reporting to private clinics. 50-70% of all TB patients prefer private healthcare, but this cohort is likely to be more heterogeneous than our sample in terms of economic backgrounds and education. Moreover, as a majority of the patients in our study were from higher socioeconomic strata, the free treatment offered by the DOTS programme may be less appealing to the patients in our study than it would be to the general population of patients.

## Conclusions

'Concordance' is defined as "the creation of an agreement between patient and healthcare provider about whether, when and how medicines are to be taken" [23]. Concordance is a cornerstone in the successful implementation of any healthcare programme, and more so in a programme such as DOTS, that demands a significant commitment from the patient in terms of time and resources.

The control of TB in India will undoubtedly benefit from more patients being attracted to and treated by the existing DOTS programme. However, our study reveals a poor knowledge and awareness of DOTS even among the better educated cohort of TB patients that we interviewed. The acceptability of DOT among private patients is an issue of "discordance" among providers and patients that needs to be addressed. DOT, in its present form, is considered too rigid and intrusive and is unlikely to be accepted by a majority of patients seeking private healthcare. Novel strategies such as family member-supervised DOTS [20] and DOTS offered through PPMs may be more flexible options that could lead to higher cure rates without compromising patient choice.

The authors declare that they have no competing interests.

## Authors' contributions

ZU conceived of the study. Both ZU and LP contributed equally to the conduct, analysis of the study, and writing of the manuscript.

## Pre-publication history

The pre-publication history for this paper can be accessed here:

http://www.biomedcentral.com/1471-2458/10/357/prepub

## Supplementary Material

Additional file 1 **Annex 3**. The original detailed questionnaire and instructions used for the study Click here for file

Additional file 2 **Table 1**. A summary of the demographic characteristics of all the participants Click here for file

Additional file 3 **Table 2**. A summary of the responses of the participants to the interview questionnaire Click here for file

Additional file 4 **Annex 1**. Abbreviated form of the questionnaire highlighting the salient questions asked Click here for file

Additional file 5 **Annex 2**. Selected unique verbatim responses from participants Click here for file

## References

[B1] Mathers CD Boerma T Ma Fat D Global and regional causes of death Br Med Bull 2009 92 7 32 10.1093/bmb/ldp028 19776034

[B2] Global tuberculosis control: surveillance, planning, financing. WHO report 2007 **World Health Organization WHO/HTM/TB/2007376**

[B3] Chadha VK Annual risk of tuberculous infection in four defined zones of India: a comparative picture Int J Tuberc Lung Dis 2005 9 5 569 75 15875931

[B4] Central TB Division, Directorate General of Health Services, Ministry of Health and Family Welfare, Government of India 2009. TB India 2009. RNTCP Status report http://www.tbcindia.org/pdfs/TB%20India%202009.pdf Accessed 1 February 2010

[B5] Uplekar M Pathania V Raviglione M Private practitioners and public health: weak links in tuberculosis control Lancet 2001 358 9285 912 6 10.1016/S0140-6736(01)06076-7 11567729

[B6] Uplekar M Rangan S Tackling TB: the search for solutions 1996 Bombay: The Foundation for Research in Community Health

[B7] Uplekar MW Shepard DS Treatment of tuberculosis by private general practitioners in India Tubercle 1991 72 4 284 90 10.1016/0041-3879(91)90055-W 1811360

[B8] Uplekar M Tuberculosis patients and practitioners in private clinics in India Int J Tuberc Lung Dis 1998 2 4 324 9 9559404

[B9] Arora VK Panel discussion on MDR and XDR TB. Moderated by Dr. V.K. Arora Indian J Tuberc 2008 55 2 104 9 18516829

[B10] Suganthi P Health seeking and knowledge about tuberculosis among persons with pulmonary symptoms and tuberculosis cases in Bangalore slums Int J Tuberc Lung Dis 2008 12 11 1268 73 18926036

[B11] Udwadia Z Specific problems of tuberculosis control in India 3 Oxford University Press

[B12] World Health Organization Anti-tuberculosis drug resistance in the world - Fourth Global Report. WHO/HTM/TB/2008.394 2008 http://www.who.int/tb/publications/2008/drs_report4_26feb08.pdf

[B13] D'Souza DT High levels of multidrug resistant tuberculosis in new and treatment-failure patients from the Revised National Tuberculosis Control Programme in an urban metropolis (Mumbai) in Western India 2009 9 BMC Public Health 211 19563647 10.1186/1471-2458-9-211 PMC2714510

[B14] Udwadia Z Pinto L Uplekar M Tuberculosis management by private practitioners in Mumbai, India: has anything changed in two decades? in press 10.1371/journal.pone.0012023 PMC2918510 20711502

[B15] Sharma N The impact of an IEC campaign on tuberculosis awareness and health seeking behaviour in Delhi, India Int J Tuberc Lung Dis 2005 9 11 1259 65 16333935

[B16] Dewan PK Improving tuberculosis control through public-private collaboration in India: literature review BMJ 2006 332 7541 574 8 16467347 10.1136/bmj.38738.473252.7C PMC1397734

[B17] Pantoja A Economic evaluation of public-private mix for tuberculosis care and control, India. Part II. Cost and cost-effectiveness Int J Tuberc Lung Dis 2009 13 6 705 12 19460245

[B18] Sudha G Factors influencing the care-seeking behaviour of chest symptomatics: a community-based study involving rural and urban population in Tamil Nadu, South India Trop Med Int Health 2003 8 4 p. 336 41 10.1046/j.1365-3156.2003.01010.x 12667153

[B19] Jaiswal A Adherence to tuberculosis treatment: lessons from the urban setting of Delhi, India Trop Med Int Health 2003 8 7 625 33 10.1046/j.1365-3156.2003.01061.x 12828545

[B20] Newell JN Family-member DOTS and community DOTS for tuberculosis control in Nepal: cluster-randomised controlled trial Lancet 2006 367 9514 903 9 10.1016/S0140-6736(06)68380-3 16546538

[B21] Lonnroth K Can I afford free treatment?: Perceived consequences of health care provider choices among people with tuberculosis in Ho Chi Minh City, Vietnam Soc Sci Med 2001 52 6 935 48 10.1016/S0277-9536(00)00195-7 11234866

[B22] Social Assessment Study for RNTCP - Final Report, Submitted to: Central TB Division, Ministry of Health and Family Welfare (Government of India), New Delhi. ORG Centre for Social Research 2003

[B23] Marinker M Shaw J Not to be taken as directed BMJ 2003 326 7385 348 9 12586645 10.1136/bmj.326.7385.348 PMC1125224

