# Supplementary material for: Private patient perceptions about a public programme; what do private Indian tuberculosis patients really feel about directly observed treatment?
Source: BMC Public Health. 2010 Jun 22;10:357. doi: 10.1186/1471-2458-10-357 (PMC2903519; doi:10.1186/1471-2458-10-357)
Supplement: Additional file 1 — Annex 3. The original detailed questionnaire and instructions used for the study [file 1471-2458-10-357-S1.PDF]

P. D. HINDUJA NATIONAL HOSPITAL
& MEDICAL RESEARCH CENTRE
(Established and managed by the National Health & Education Society)

VEER SAVARKAR MARG, MAHIM, MUMBAI - 400 016, INDIA
PHONE : 2445 1515, 2445 2222, 2444 9199 FAX : 2444 9151

PRIVATE TB PATIENT'S PERCEPTION OF DOTS

I. Patient demographics

(a) **Name :**

(b) **Age :**

(c) **Sex : Male** ☐ **Female** ☐

(d) **Marital Status : Single** ☐ **Married** ☐

(e) **Type of Family :**

Living alone ☐ **Nuclear** ☐ **Joint** ☐

(f) **Education : Illiterate** ☐ **Primary** ☐

Secondary ☐ **Graduate** ☐

Post Graduate ☐

(g) **Occupation : Skilled** ☐ **Semiskilled** ☐

Unskilled ☐ **Unemployed** ☐

Student ☐ **Retired** ☐

Housewife ☐

(h) Family Income (Rs / month) :

< 5000 ☐ 5001 – 10,000 ☐ 10,001 – 25,000 ☐

25001 – 50,000 ☐ > 50,000 ☐

II Patient's Perception of DOTS :

(i) Have you heard of the new strategy to fight TB called DOTS ?

Y ☐ N ☐

(ii) If yes , what is the full form of DOTS ? - - - -

(iii) Do you have any idea what it means or involves ?

The patient at this stage is given information regarding DOTS (information accessed from www.tbcindia.org) – the next two pages are read out / handed over to the patient, in a language he/she understands, and any clarification sought is explained.

Directly Observed Treatment, Short-course (DOTS)

The DOTS strategy along with the other components of the Stop TB strategy, implemented under the Revised National Tuberculosis Control Programme (RNTCP) in India, is a comprehensive package for TB control.

The DOTS strategy is cost-effective and is today the international standard for TB control programmes. To date, more than 180 countries are implementing the DOTS strategy. India has adapted and tested the DOTS strategy in various parts of the country since 1993, with excellent results, and by March 2006 nationwide DOTS coverage has been achieved.

DOTS is a systematic strategy which has five components

- **Political and administrative commitment.** TB is the leading infectious cause of death among adults. TB kills more men than women, yet more women die of TB than all causes associated with childbirth combined. Since TB can be cured and the epidemic reversed, it warrants the topmost priority, which it has been accorded by the Government of India. This priority must be continued and expanded at the state, district and local levels.

- **Good quality diagnosis.** Good quality microscopy allows health workers to see the tubercle bacilli and is essential to identify the infectious patients who need treatment the most.

- **Good quality drugs. An uninterrupted supply of good quality anti-TB drugs** must be available. In the RNTCP, a box of medications for the entire treatment is earmarked for every patient registered, ensuring the availability of the full course of treatment the moment the patient is initiated on treatment. Hence in DOTS, the treatment can never interrupt for lack of medicine.

- **Supervised treatment to ensure the right treatment**, given in the right way. The RNTCP uses the best anti-TB medications available. But unless treatment is made convenient for patients, it will fail. This is why the heart of the DOTS programme is "directly observed treatment" in which a health worker, or another trained person who is not a family member, watches as the patient swallows the anti-TB medicines in their presence.

- **Systematic monitoring and accountability.** The programme is accountable for the outcome of every patient treated. This is done using standard recording and reporting system, and the technique of 'cohort analysis'. The cure rate and other key indicators are monitored at every level of the health system, and if any area is not meeting expectations, supervision is intensified. The RNTCP shifts the responsibility for cure from the patient to the health system.

(iv) Do you find this form of treatment acceptable ?

Y ☐ **N** ☐

(v) If yes, give reasons :

(vi) If no, give reasons :

(vii) Would you be prepared yourself to visit a TB clinic as frequently as three times a week to receive your drugs ?

Y ☐ **N** ☐

(viii) Would you prefer buying the drugs yourself once a month ?

Y ☐ **N** ☐

(ix) Would you be prepared to have a doctor or nurse actually observe you swallow each dose of your TB drugs under superivision ? **Y** ☐ **N** ☐

If no why ?

(x) Why did you choose to see a private TB specialist instead of going to a government TB clinic ?

(xi) How would you rate the government TB services and what do you think are their weaknesses ?

(xii) How many doctors in all (include GPs, Homeopaths & Ayurveds) have you seen for your TB before you consulted the clinic at P.D.Hinduja National ahOSPITAL AND Medical Research Centre ?

Number

Type of Doctor
