# Supplementary material for: Private patient perceptions about a public programme; what do private Indian tuberculosis patients really feel about directly observed treatment?
Source: BMC Public Health. 2010 Jun 22;10:357. doi: 10.1186/1471-2458-10-357 (PMC2903519; doi:10.1186/1471-2458-10-357)
Supplement: Additional file 5 — Annex 2. Selected unique verbatim responses from participants [file 1471-2458-10-357-S5.DOC]

**ANNEX 2:**

“The line of treatment is very important for people who are illiterate and who live below the poverty line, but not for me”

“Directly observed treatment is useful for careless people because the patient cannot miss the drug”

“This type of treatment would serve well for the lower middle class person who may not be able to afford a proper diagnosis and treatment”

“I can’t trust the government for such a killer disease”

“I am educated and full aware of consequences. I can supervise myself and take medicines regularly as advised”

“It is a very kiddish method”

“I am quite aware of my illness. I am cautious and if I want to get better I would take the medicine with devotion.”

“I would prefer to be treated privately and I am educated enough to understand the consequences of not taking the proper doses on time. Hence I’d like to buy my own medication”

“We are in a joint family, my wife and brother care for me. They make sure I take drugs regularly”

“I would like to have some privacy as I am a private person”

“I can’t risk my life as government hospitals are too unreliable”

“Government is always government!”
